# Solvent-mediated crystallization and defect passivation mechanisms in ambient-air MAPbI_3_ films: a combined experimental and simulation study

**DOI:** 10.1039/d5ra03835a

**Published:** 2025-07-03

**Authors:** Ichrak Touhami, Ouidad Beldjebli, Selma Rabhi, Yaacoub Ibrahim Bouderbala, Béchir Dridi Rezgui, Tarak Hidouri, Amal BaQais, Shima Sadaf, Mir Waqas Alam, Mongi Bouaïcha

**Affiliations:** a Université de Tunis El Manar Campus Universitaire Farhat Hached, B.P. No. 94, Rommana 1068 Tunis Tunisia ichrak.touhami@etudiant-fst.utm.tn; b Laboratoire de Photovoltaïque, Centre de Recherches et des Technologies de l'Energie Technopôle de Borj-Cédria, B.P. No. 95, Hammam-Lif 2050 Tunis Tunisia; c Laboratoire de Chimie Appliquée et Génie Chimique, Université Mouloud Mammeri B.P. No. 17 RP 15000 Tizi-Ouzou Algeria; d Laboratory of Innovative Environmental Preservation Techniques, Department of Chemistry, Constantine 1 University 25000 Constantine Algeria selma.rabhi@umc.edu.dz; e Applied Optics Laboratory, Institute of Optics and Precision Mechanics, University of Ferhat Abbas 19000 Setif Algeria; f Department of Mathematical, Physical and Computer Sciences, University of Parma 43124 Parma Italy tarek.hidouri@unipr.it; g Department of Chemistry, College of Science, Princess Nourah Bint Abdulrahman University Riyadh 11671 Saudi Arabia; h Department of Electrical Engineering, College of Engineering King Faisal University Al-Ahsa 31982 Saudi Arabia ssadaf@kfu.edu.sa; i Department of Physics, College of Science, King Faisal University Ahsaa 31982 Saudi Arabia

## Abstract

Understanding solvent-induced crystallization and defect dynamics in hybrid perovskites is crucial for stable and high-efficiency solar cells. Here, we investigate the influence of DMF : DMSO solvent ratios on the nucleation, grain boundary passivation, and optoelectronic behaviors of MAPbI_3_ thin films prepared under ambient conditions. X-ray diffraction and photoluminescence analyses reveal that increasing DMSO content promotes PbI_2_ segregation, which passivates grain boundaries and modulates trap-assisted recombination. These structural and photophysical changes are correlated with carrier lifetimes using time-resolved photoluminescence measurements and the simulated device performance using SCAPS-1D. We find that a DMF : DMSO ratio of (6 : 4) enhances the interplay between crystalline order, the energy disorder (Urbach tail), and the charge transport. Our findings provide mechanistic insights into how solvent coordination controls defect landscapes and interface energetics in perovskite films, offering a framework for rational solvent design in ambient-stable photovoltaics.

## Abstract 2

Understanding how solvent composition influences crystallization behavior and defect formation in hybrid perovskites is essential for optimizing film quality, enhancing structural stability, and achieving high-efficiency and durable perovskite solar cells.

## Graphical abstract

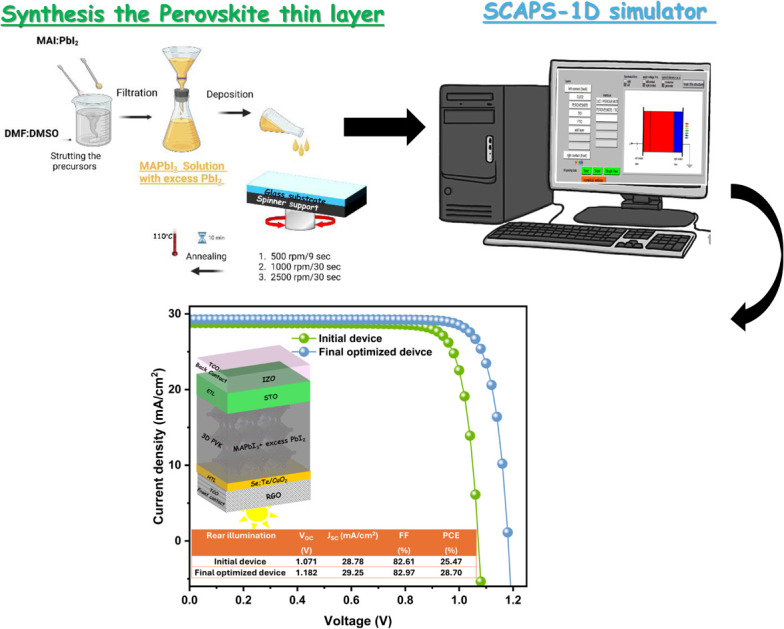

## 1. Introduction

**Fig. 1:**
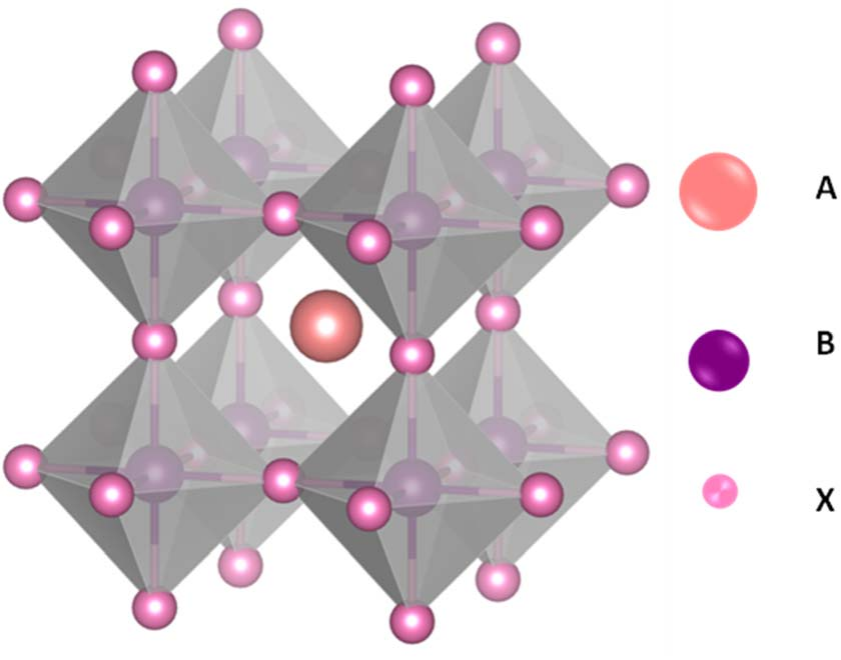
Perovskite crystalline structure realised by VESTA.

Solar energy is a leading renewable resource, offering abundant, clean, and eco-friendly power.^1^ This limitation has spurred significant interest in the development of new materials and alternative strategies for clean energy generation. Among these, perovskite materials have attracted considerable attention due to their exceptional optoelectronic properties and unique ABX_3_ crystal structure, where A is a monovalent cation, which can be an organic cation such as methylammonium (MA^+^) or formamidinium (FA^+^), an inorganic cation like cesium (Cs^+^), or a mixture of two or more of these cations to improve the stability and performance of the perovskite material. B is a divalent metal cation (commonly lead (Pb^2+^) or tin (Sn^2+^), and X is a halide anion (such as iodide (I^−^), bromide (Br^−^), or chloride (Cl^−^)). This structure forms a three-dimensional lattice in which the B-site cation is octahedrally coordinated by six X-site anions, and the A-site cation fills the voids between the octahedra as presented in Fig. 1. The flexibility in tuning the A, B, and X components allows precise control over the material's electronic, optical, and structural properties.

Beyond their widely studied application in perovskite solar cells (PSCs), these materials also show great promise in a variety of other fields, including photocatalysis,^2–4^ light-emitting diodes (LEDs), lasers, and photodetectors,^5,6^ making them highly versatile candidates for next-generation energy and optoelectronic technologies. PSCs have quickly become a game-changing technology in the solar energy industry. A direct band gap, low exciton binding energy, excellent light absorption capabilities, and inexpensive manufacture are some of their many strong points.^7^ The potential of PSCs was first demonstrated in 2009 by Kojima *et al.*,^8^ who achieved a modest PCE of 3.8% using methyl ammonium lead iodide (MAPbI_3_) as the active layer. Since then, the field has advanced rapidly, with current efficiencies exceeding 26.7%,^9^ positioning PSCs as a leading candidate for next-generation PV technologies. However, their commercial viability remains hindered by intrinsic instability and defect mediated recombination. To address these challenges, researchers have increasingly focused on solvent engineering and defect passivation, particularly at the perovskite-hole transport layer (HTL) interface. Passivating defects at the perovskite/HTL (hole transport layer) interface by incorporating an excess PbI_2_ is considered as one of those approaches.^10,11^ This passivation reduces non-radiative recombination, thereby improving both efficiency and stability, as extensively reported in the literature. Here, dimethyl sulfoxide (DMSO) plays a pivotal role: its strong coordination with Pb^2+^ ions modulated solubility and crystallization kinetics, enabling the formation of uniform, large-grained perovskite films with tailored PbI_2_ distribution.^12–15^ Fabrication techniques also play a pivotal role in determining the quality and stability of PSCs. These techniques can generally be categorized into dry and wet processes, with the latter being more cost-effective and widely adopted in PSC manufacturing.^16–19^ Among wet processes, spin-coating is a frequently employed deposition method for fabricating MAPbI_3_ thin films. This method is implemented *via* two main approaches: the one-step and two-step deposition methods.^20^ The one-step method involves spin-coating a precursor solution containing CH_3_NH_3_I/MAI and PbI_2_, followed by annealing the films at temperatures alternating from 70 °C to 150 °C to crystallize the perovskite layer. This method is favored for its simplicity, cost-effectiveness, and suitability for large-scale production. In contrast, the two-step method involves initially spin-coating of a PbI_2_ film, followed by immersing it in a CH_3_NH_3_I solution to facilitate the formation of a MAPbI_3_ layer upon annealing. While this approach allows for greater control over film formation and facilitates the rapid fabrication of PSCs, it is often limited by the difficulty in controlling the fast crystallization of MAPbI_3_ throughout the procedure, particularly in ambient-air condition.^21,22^ To optimize PSC performance, analyzing the physical properties of MAPbI_3_ films is an essential process for achieving optimal device structures. Eventually, cooperation between solvent engineering, interface passivation and advanced fabrication protocols is key to fabricating high-potential perovskite solar cells. In this work, we combine experimental data of MAPbI_3_ including lifetime, bandgap, and thickness, with SCAPS-1D simulations to create an efficient protocol for accelerating the fabrication of perovskite solar cells. This approach not only saves time and resources but also provides a valuable framework for optimizing device performance. In plus, we propose the simulation of inverted perovskite solar cells, which have demonstrated higher stability compared to conventional perovskite solar cells. Although the power conversion efficiency of p-i-n structures remains lower than that of n-i-p devices, we suggest a new configuration incorporating novel hole transport layers, electron transport layers, and transparent conductive oxides to enhance photovoltaic performance.

## 2. Experimental and simulations details

### 2.1 Fabrication and characterization of MAPbI_3_ films

**Fig. 2:**
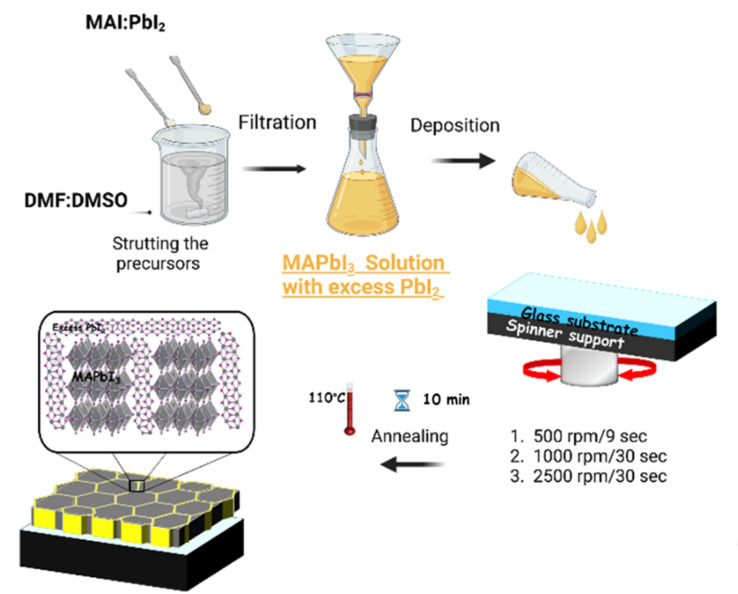
Overview schematics of MAPbI_3_ films fabrication procedures.

MAPbI_3_ films were spin-coated on glass substrates, *via* a one-step process, using four solutions with different DMF : DMSO ratios of 1 : 0, 8 : 2, 6 : 4 and 5 : 5. The samples obtained were named as S1, S2, S3 and S4, respectively for DMF : DMSO ratios of 1 : 0, 8 : 2, 6 : 4 and 5 : 5. Fig. 2 shows a schematic of the synthesis procedure of MAPbI_3_ highlighting the PbI_2_ segregation on it. Indeed, the solutions were prepared by dissolving 1 mol of methyl ammonium iodide (MAI) and 3 mol of lead iodide (PbI_2_) in 2 mL of a solvent mixture containing the mentioned ratios of DMF : DMSO. These solutions were stirred at 60 °C for 4 h to obtain homogeneous solutions. Then, they were spin-coated on the glass substrates with three successive rotation speeds: 500 rpm for 9 s, 1000 rpm for 30 s, and then 2500 rpm for 30 s. Finally, the four samples were annealed under ambient conditions at 110 °C for 15 min, to promote the crystallization of MAPbI_3_.

The crystalline structure of the samples was identified by X-ray diffraction (XRD) using a Bruker D8 Advance diffractometer in Bragg–Brentano mode with CuK_α_ radiation (*λ* = 1.54 Å). The transmittance and absorption spectra were collected using a monochromator-based PTS-2-QE/IPCE spectrophotometer. The room-temperature steady-state photoluminescence (PL) spectra were measured using a Jobin-Yvon HR 250 spectrophotometer using an excitation laser source of *λ* = 447 nm. The PL lifetime (TRPL) was measured by Edinburgh FLS980 spectrometer (Xe lamp/RCSPC) equipped with a Red PMT detector. The repetition rate is fixed at 4 MHz with a time range of 200 ns.

### 2.2 Simulation of perovskite solar cell

We proceed to analyze the MAPbI_3_-based PSC using the physical properties of MAPbI_3_ extracted experimentally and additional integration of theoretical/simulation approaches through the Solar Cell Capacitance Simulator SCAPS-1D version 3.3.10.^23–27^ The simulation was performed simultaneously based on a traditional drift-diffusion model and solving the electron and hole continuity eqn (1) and (2) as well as the Poisson's eqn (3).1
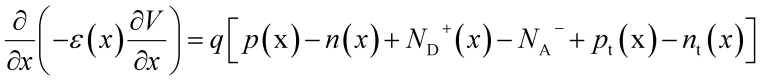
2
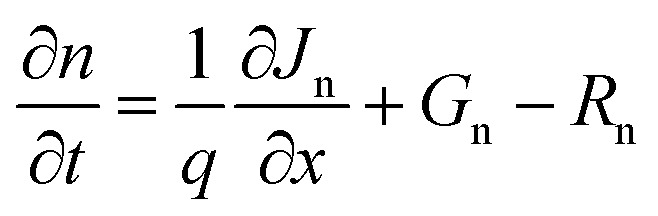
3
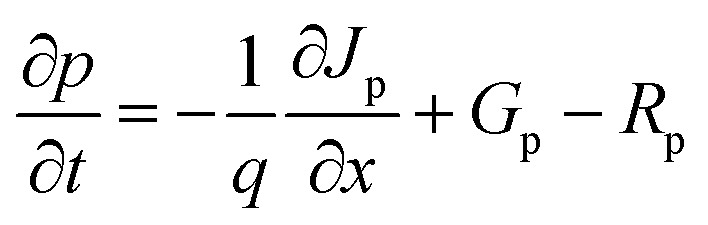
where *ε*, *V*, *q*, *p*(*x*), *n*(*x*), *N*_D_^+^(*x*), *N*_A_^−^(*x*), *p*_t_(*x*), and *n*_t_(*x*) are dielectric permittivity, electric potential, electronic charge, free hole density, free electron density, donor density, acceptor density, trap density of holes, and trap density of electrons, respectively (Table 1).

**Table 1:** Parameters used for TCO (IZO), 3D-PVK (MAPbI_3_), and organic–inorganic HTLs and ETLs of the studied IPSCs, where *E*_g_: the band gap, *χ*: the electron affinity, *ε*: the dielectric permittivity, *C*_b_: the conduction band effective density of states, *V*_b_: the valence band effective density of states, *μ*_e_: the electron mobility, *μ*_n_: the hole mobility, *N*_a_: the shallow uniform acceptor density, *N*_d_: the shallow uniform donor density, *N*_t_: the defect density

	TCO	3D-PVK	HTLS					ETLs	
Parameters	IZO	MAPbI_3_	CBTS	Me-4PACz	PATAA	NiO	Se/Te:Cu_2_O	STO	MZO
Thickness (μm)	a	a	a	a	a	a	a	a	a
*E*_g_ (eV)	3.5	a	1.9	3.3	2.95	3.8	1.88	3.2	3.35
*χ* (eV)	4.5	3.88	3.6	2.8	2.3	1.4	3.53	4	4
*ε*	10	24.1	5.4	10	3.5	10.7	7.11	8.7	66
*C*_b_ (cm^−3^)	1 × 10^19^	8.1 × 10^18^	2.2 × 10^18^	1 × 10^19^	2 × 10^21^	2 × 10^19^	2.02 × 10^17^	1.7 × 10^19^	1 × 10^19^
*V*_b_ (cm^−3^)	1 × 10^19^	8.1 × 10^18^	1.8 × 10^19^	1 × 10^19^	2 × 10^21^	1.8 × 10^19^	1.17 × 10^19^	2 × 10^20^	1 × 10^19^
*μ*_e_ (cm^2^ V^−1^ s^−1^)	1 × 10^−2^	1	30	6	1 × 10^−4^	12	1297	5.3 × 10^3^	0.05
*μ*_n_ (cm^2^ V^−1^ s^−1^)	1 × 10^−3^	1	10	24	1 × 10^−4^	28	1297	6.6 × 10^2^	0.05
*N*_a_ (cm^−3^)	1 × 10^18^	1 × 10^14^	0	0	0	0	0	2 × 10^16^	1 × 10^17^
*N*_d_ (cm^−3^)	—	1 × 10^14^	1.1 × 10^15^	1 × 10^18^	1.0 × 10^18^	5 × 10^19^	3.0 × 10^18^	0	0
*N*_t_ (cm^−3^)	1 × 10^14^	a	1.0 × 10^14^	a	1.0 × 10^14^	1 × 10^14^	1.0 × 10^15^		1 × 10^14^
Reference	28		29	30	31	32	33	34	35

a This study.

## 3. Results and discussion

### 3.1 Structural and optical analysis

Fig. 2 displays the XRD pattern of MAPbI_3_ films prepared with different DMF : DMSO ratios, maintaining the same excess of PbI_2_ all the samples. A mixture of both the tetragonal phase of MAPbI_3_ and the hexagonal phase of PbI_2_ was observed for all films. The tetragonal phase of MAPbI_3_ can be verified by the existence of (110), (112), (211), (202), (220), (222), (312), (224), (330) and (404) planes.^10,36^ All the films display a polycrystalline nature with a preferred orientation along the (110) plane. However, the peaks at 12.60° and 37.83° are attributed to the (001) and the (003) crystal planes of the hexagonal structure of PbI_2_, confirming the presence of residual PbI_2_ excess in all samples. Because we use an excess solution to prepare the films, it is expected that PbI_2_ will appear as the dominant phase in comparison to the main perovskite phase. This residual PbI_2_ plays a beneficial role in forming a passivation layer that enhances the interface between the perovskite layer and the HTL, improving the charge transport and reducing recombination losses.^11,37^

**Fig. 3:**
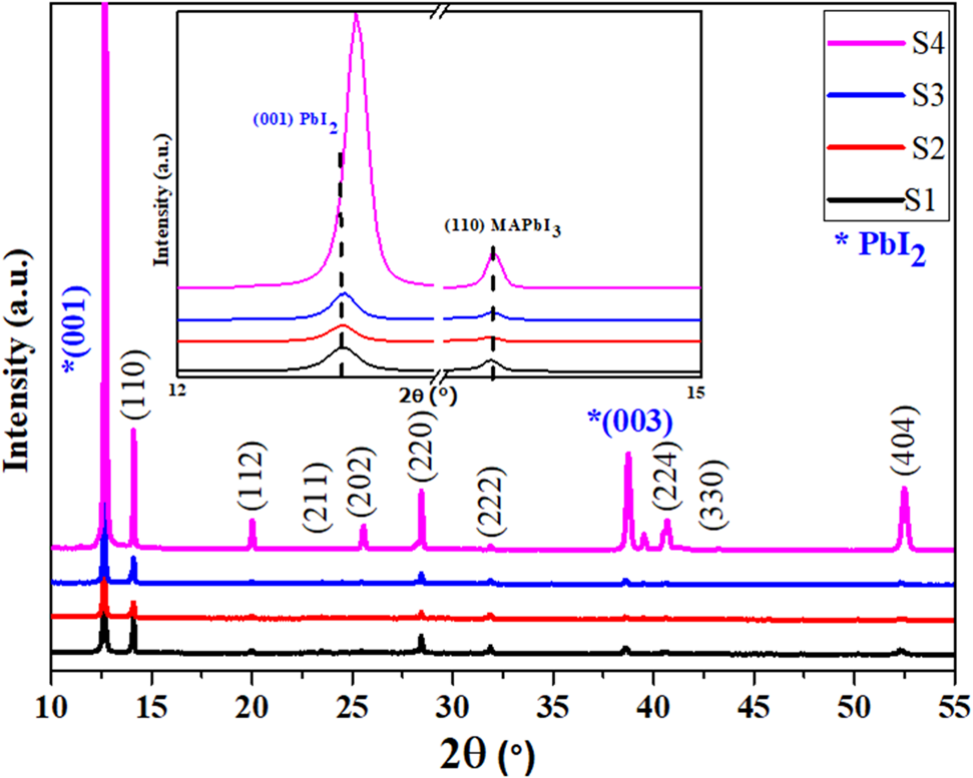
X-ray diffraction spectra of perovskite layers for different DMF : DMSO ratios.

On the other hand, Fig. 3 shows that there is no shift in peak positions related to the MAPbI_3_ tetragonal phase. This is reflecting the fact that DMF : DMSO ratios do not influence the lattice parameters (*a* and *c* lattice parameters). In contrast, with raising DMSO content up to 50%, a slight shift of the (001) plan related to the PbI_2_ hexagonal phase towards higher diffraction angles is observed. This result suggests a lattice contraction within the PbI_2_ structure by decreasing their lattice parameters (*a* and/or *c* lattice parameters). The (001) peak shift highlights the DMSO effective role in ambient-air processing since it coordinates with Pb^2+^ ions during crystallization. Indeed, it form an intermediate PbI_2_-DMSO complexes that delay the crystallization and allow for more ordered packing of PbI_2_ layers, resulting in a more stable perovskite film.

The lattice parameters *a* and *c* of the MAPbI_3_ tetragonal phase of our samples were determined using Bragg's law (eqn (4) and (5)). The obtained values are displayed in Table 2.

4 2*d* sin *θ* = *nλ*

5
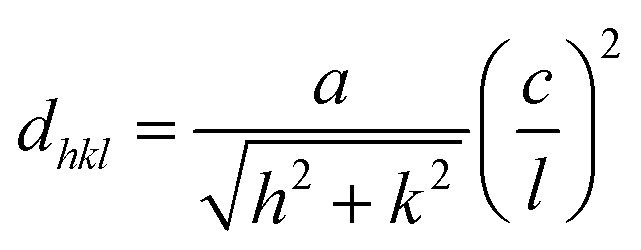
where *d* is the interplanar distance, *λ* (CuKα) is the wavelength of the incoming copper radiation (1.5406 Å), and *θ* is the diffraction angle corresponding to the Miller indices *h*, *k*, and *l*, respectively.

**Table 2:** DMF : DMSO ratio, structural parameters, thicknesses, band gap energy (*E*_g_), and disorder energy (*E*_u_) of the prepared perovskite films

Samples	S1	S2	S3	S4
DMF : DMSO ratio	1 : 0	8 : 2	6 : 4	5 : 5
Lattice parameters of MAPbI_3_ phase (Å)	*a* = 8.8688	*a* = 8.8691	*a* = 8.8679	*a* = 8.8622
	*b* = 8.8688	*b* = 8.8691	*b* = 8.8679	*b* = 8.8622
	*c* = 11.3564	*c* = 11.3548	*c* = 11.3593	*c* = 11.3553
(110)_MAPbI_3__/(001)_PbI_2__	0.51	0.3	0.32	0.14
FWHM of (001)-PbI_2_	0.120	0.120	0.096	0.12
FWHM of (110)-MAPbI_3_	0.072	0.072	0.096	0.072
*D* (nm) of MAPbI_3_	104.67	99.7	94.8	86.6
*D* (nm) of PbI_2_	61.33	68.51	72.19	82.80
*ε* of MAPbI_3_	0.18	0.19	0.2	0.23
Thicknesses (nm)	810	820	813	800
*E*_g_ (eV)	1.48	1.43	1.46	1.50
*E*_u_ (meV)	154.5	288.5	206	115
Lifetime average *τ*_avg_ (ns)	768.52	741.83	845.9	765.3

From Table 2, the lattice parameters of MAPbI_3_ tetragonal phase of all the samples are around 8.87 Å and *c* = 11.35 Å, confirming that the DMF : DMSO ratios have no effect. It is reported that the range of the bulk values of *a* and *c* are from 8.83 Å to 8.90 Å, and from 12.60 Å to 12.68 Å, respectively.^38^ In comparison, our measured *c* value is lower than these later, meaning that the films are subject to a compressive stress formed during solvent evaporation and annealing treatment. Furthermore, by examining the intensity of peaks from Fig. 3, it can be observed that the intensity of all the XRD peaks increases for the (5 : 5) DMF : DMSO ratio suggesting an enhanced crystallinity. This improvement is attributed to more controlled crystallization dynamics where the presence of DMSO facilitates the formation of intermediate PbI_2_·DMSO complexes that slow down solvent evaporation and promote gradual nucleation.^39^ As a result, this sample exhibits smaller MAPbI_3_ crystallites (86.6 nm) and larger PbI_2_ grains (82.8 nm), as reported in Table 2. The growth of larger PbI_2_ crystallites is particularly beneficial, as it correlates with improved defect passivation at grain boundaries. This is supported by the decrease in the (110)MAPbI_3_/(001)PbI_2_ peak intensity ratio from 0.51 to 0.14 with increasing DMSO content (see Table 2), confirming the enhanced PbI_2_ crystallization.^40^ In contrast to mixed solvent systems, the film prepared using pure DMF (1 : 0) exhibits the largest MAPbI_3_ crystallite size (104.67 nm) and the smallest PbI_2_ crystallites (61.33 nm), indicating rapid and uncontrolled crystallization. This behavior results from the fast evaporation of DMF, which hinders the formation of intermediate phases that are essential for controlled nucleation and effective defect passivation. As the DMSO content increases, a gradual decrease in MAPbI_3_ crystallite size is observed from 104.7 nm to 86.6 nm accompanied by an increase in PbI_2_ grain size. This trend highlights the role of solvent coordination in modulating crystallization dynamics. Specifically, the formation of PbI_2_·DMSO complexes slows down the crystallization process, allowing for more uniform grain growth and improved film morphology.

Additionally, the strain (*ε*) in the perovskite lattice increases with DMSO content, from 0.18 in S1 to 0.23 in S4. This may be linked to the interfacial stress caused by larger PbI_2_ inclusions. These XRD-based observations confirm that while high DMSO content promotes crystallinity and passivation, it also introduces residual strain, highlighting the importance of tuning solvent composition.

For further explanation, the average (*D*) of crystallite sizes and the strain (*ε*) in films were calculated by the Scherrer and Williamson–Hall methods (eqn (6)) and (eqn (7)), from the XRD pattern.^41^ The values were recorded in Table 2.6
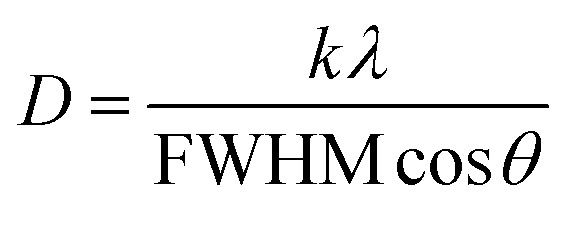
7
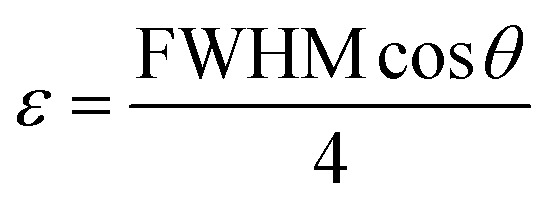
where *D* is the crystallite size (nm), *λ* (CuK_α_) is the wavelength of copper incident radiation (1.5406 Å), *k* = 0.94 is a constant, *θ*(°) is the Bragg angle, and the FWHM (Rad) represents the average full width at half maximum of the selected diffraction peaks. In this study, the crystallite sizes of the perovskite and residual PbI_2_ phases were calculated using the Debye–Scherrer equation applied to selected XRD peaks. Specifically, the (110) peak of tetragonal MAPbI_3_ and the (001) peak of hexagonal PbI_2_ were chosen, as they are the most intense and representative reflections for each phase. These dominant peaks offer reliable indicators of crystal growth and structural evolution. FWHM values used in these calculations are labeled in Table 2 with the corresponding average crystallite sizes.

**Fig. 4:**
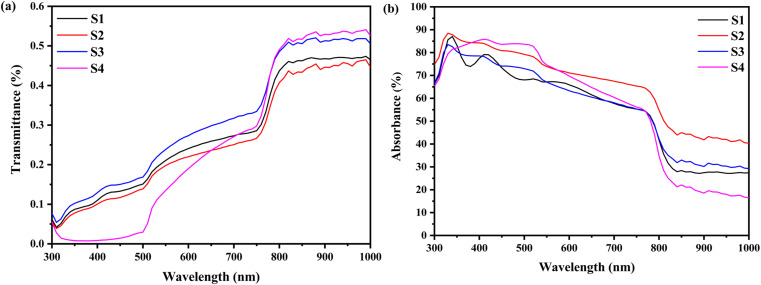
(a) Optical transmittance spectra, (b) absorbance spectra of MAPbI_3_ films with different DMF : DMSO ratios of samples S1, S2, S3 and S4.

The optical transmittance spectra of MAPbI_3_ films with different DMF : DMSO ratios, are shown in Fig. 4a. These spectra were used to calculate the film thicknesses by the envelope method.^42^ The envelope method is based on analyzing the interference fringes that appear in the optical transmittance spectra (Fig. 4a), which result from constructive and destructive interference of light within the film. These fringes arise from multiple reflections at the air/film and film/substrate (glass) interfaces. In our study, all MAPbI_3_ films exhibited clear oscillatory behavior, indicating sufficient film thickness and optical smoothness to allow interference effects. This technique enables the determination of both the wavelength-dependent refractive index *n*(*λ*) and the film thickness *d* without requiring cross-sectional scanning electron microscope (SEM) imaging. Therefore, it serves as a non-destructive and effective approach for characterizing thin films deposited on transparent substrates. Using the Swanepoel method and the eqn (8), the refractive index is determined by utilizing the transmittance envelope maxima (*T*_max_) and minima (*T*_min_).^42^8
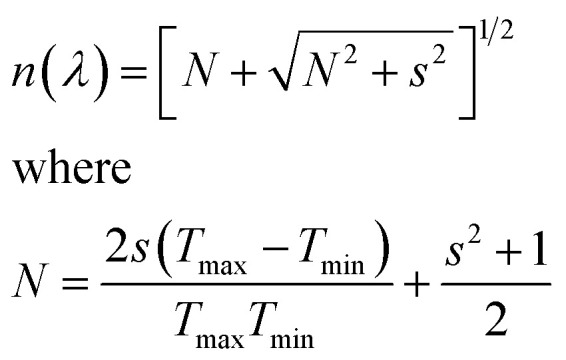


The factor *s* is the refractive index of the glass substrate (typically *s* ≈ 1.51). By applying the standard envelope method formula (eqn (9)), the film thickness (*d*) can be estimated without physical cross-sectioning.^42^9
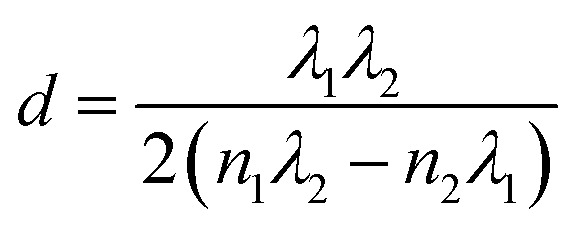
where *λ*_1_ and *λ*_2_ are wavelengths of two successive maxima (or minima), and the corresponding refractive indexes *n*_1_ and *n*_2_.

This method is especially suitable for semi-transparent and uniform thin films deposited on transparent substrates. The calculated film thickness values obtained from this approach are reported in Table 2. Furthermore, in the visible range, the films displayed a low transmittance of less than 0.3%, due to their high absorbance. We also noted an absorption edge at around 750 nm, corresponding to the optical band gap of the MAPbI_3_ films.

Besides, the absorption spectra of MAPbI_3_ films are shown in Fig. 4b. All the samples exhibited a high absorbance covering a broad range of light from the ultra-violet to the near-infrared region. Samples S2 and S4 samples represent the higher absorbance values. These results suggest the strong light absorption of our perovskite films, which works as the solar cells' active layer, causes the higher short-circuit current density.

**Fig. 5:**
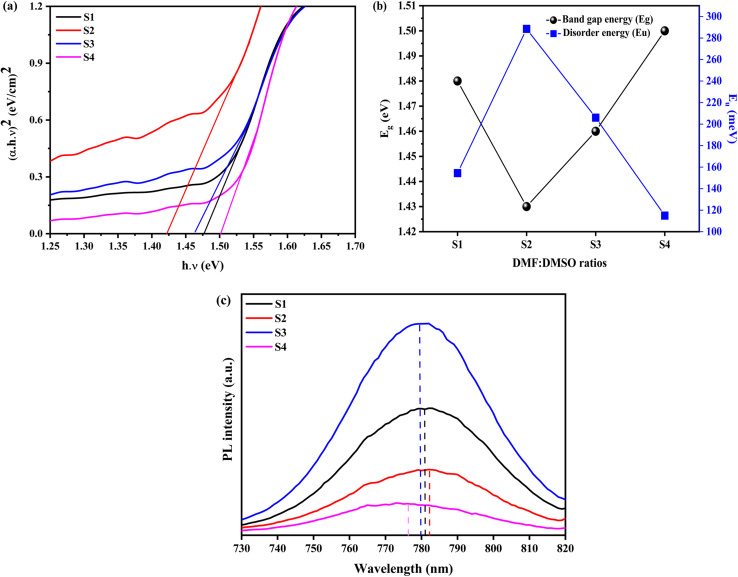
(a) Tauc plots, (b) band gap energy and disorder energy. (c) PL spectra of S1, S2, S3 and S4.

Furthermore, the absorption edge of the films slightly shifts to lower wavelengths as the DMSO content increases. It mainly arise from the nanoscale structural (in the present case, it is due to the reduction in the crystallite size of MAPbI_3_ film). This reflects an increase in the optical band gap (*E*_g_). The Tauc formula (eqn (10)) is used to compute it:

10 (*αhν*)^2^ = *A*(*E*_g_ − *hν*)

where *α* is the absorption coefficient, *hν* is the photon energy, *h* is Planck's constant, and *A* is a constant. Fig. 5a shows the plot of (*αhν*)^2^*versus* the incident energy (*hν*). The estimated values of *E*_g_ were obtained by the extrapolation of the straight line to *α* = 0. Furthermore, the disorder in the MAPbI_3_ films is described by the band tail width which is called Urbach tail and given by the relation:11
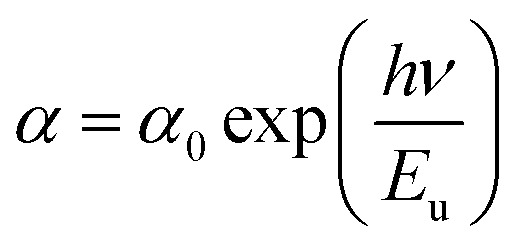
where *α*_0_ the pre-exponential factor, and *E*_u_ is the Urbach tail (disorder energy). The latter can be estimated from the inverse slope of the linear plot of ln(*α*) *versus* photon energy.

Both energy band gap and disorder energy (Urbach tail) are represented as a function of DMF : DMSO ratios in Table 2 and Fig. 5b. As can be seen, the variations of these two parameters have opposite trends. The variation in the optical band gap (*E*_g_) of MAPbI_3_ films with different DMF : DMSO ratios is primarily attributed to changes in crystallite size and structural disorder. As the DMSO content increases from 0% to 20%, *E*_g_ decreases from 1.48 eV to 1.43 eV due to the formation of intermediate PbI_2_·DMSO complexes, which promote better crystallization and reduce energetic disorder.^43^ This is clear from the increased Urbach energy (*E*_u_), indicating a higher density of localized states that narrows the apparent band gap.^44^ However, as the DMSO content increases further (up to the 5 : 5 ratio), *E*_g_ rises to 1.50 eV.^45^ This is associated with a reduction in crystallite size and a decrease in disorder (*E*_u_ = 115 meV), which suggests improved structural ordering and fewer sub-bandgap states. Additionally, the smaller grain size at higher DMSO content may introduce mild quantum confinement effects, contributing to the widening of the band gap. These results confirm that both crystallite size and energetic disorder, modulated by solvent ratio, play a critical role in tuning the band gap of MAPbI_3_ films processed under ambient conditions.

However, other samples possess higher disorder energy implying that this sample displays the most significant structural disorder, which is confirmed by its lowest XRD peak intensity. The elevated density of localized states within the band gap may stem from a significant concentration of lattice defects, grain boundaries, or impurities, resulting in enhanced optical absorption in the sub-bandgap region. The high absorbance from the ultra-violet to the near-IR region, with a band gap in the range of 1.43–1.50 eV of the MAPbI_3_ thin films, proves their potential as an effective absorber layer in solar cells.

Fig. 5c illustrates the PL spectra of MAPbI_3_ films prepared with varying DMF : DMSO ratios and deposited on glass substrates. All films display a characteristic red emission peak in the visible range attributed to the radiative recombination of the photogenerated carriers across the MAPbI_3_ bandgap. Specifically, the red band ‘maxima is located around 781.19 nm (1.58 eV), 782.12 nm (1.58 eV), 780.26 nm (1.59 eV), and 774.48 nm (1.60 eV) correspond to 1 : 0, 8 : 2, 6 : 4 and 5 : 5 DMF : DMSO ratios, respectively. These values align closely with the optical bandgap energies derived from UV-Vis measurements (Table 2), confirming the intrinsic electronic transitions between the valence band (VB) and the conduction band (CB) of MAPbI_3_.^46–48^ In addition, the narrow symmetric PL profiles further suggest better material quality with minimal defects and a well-crystallized perovskite film. Notably, the blueshift of the PL peak position with increasing DMSO content can be attributed to the formation of an intermediate adduct phase during crystallization, inducing lattice strain that slightly widens the bandgap. In plus, it could be assigned to the growth of PbI_2_ at the grain boundaries that itself perturb the perovskite's electronic structure. Furthermore, the reduction of crystallites size of MAPbI_3_ can introduce a quantum confinement effect that amplifies the bandgap could be another reason for the observed blueshift. As shown in Fig. 5c, the PL intensity increases when the DMSO content increases from 0 to 20%, peaking at the 8 : 3 DMF : DMSO ratio, then it decreases as a function of DMSO content, and reaches a minimum value for 5 : 5 DMF : DMSO ratio. The rise in the PL peak intensity with 20% DMSO indicates an improved crystal phase formation and increased electron–hole recombination pathways; as DMSO facilitates intermediate adduct phase formation during deposition, enabling uniform crystal growth with fewer defects. However, beyond 20% DMSO, the PL intensity decreases due to the reduction of the radiative recombination rate of MAPbI_3_ films. The excessive DMSO in sample S4 destabilizes the adduct decomposition during annealing by extending solvent retention leading to an incomplete perovskite conversion, an amorphous PbI_2_ aggregation, or disordered MAPbI_3_ growth. In addition, the residual DMSO solvent can lead to the creation of halide vacancies or charge traps by acting as a Lewis base. The decline coincides with excess DMSO and lead diiodide, as confirmed by XRD analysis. Therefore, while the excessive PbI_2_ treatment was found to passivate grain boundaries, and suppress the formation of trap states in the perovskite material, enhancing charge carrier movement and decreasing electron–hole recombination rate,^49^ its overuse introduces new defects that counteract its benefits.

The latter improves carrier dynamics and the overall optoelectronic performance of the MAPbI_3_ films by facilitating more efficient charge carrier extraction and transport, which is critical for applications in photovoltaic and optoelectronic devices. The PL analysis is in good agreement with XRD and optical (UV-Vis) data.

In the photovoltaic field, solar cells operate following three processes: charge carrier generation, separation and then collection. Among these, maximizing charge collection is one of the overarching goals of these cells. To elucidate the interplay between material properties and carriers, we conducted time resolved photoluminescence (TRPL) measurement. This technique quantifies carrier lifetimes, offering insights into recombination kinetic and defect-mediated losses.

**Fig. 6:**
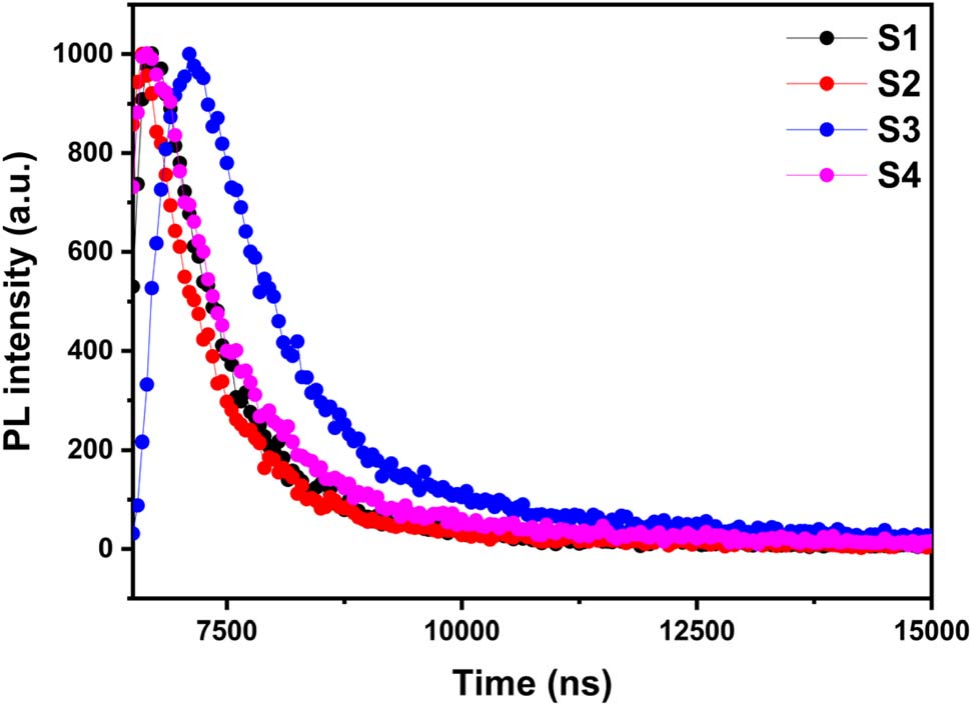
TRPL spectra of MAPbI_3_ films prepared with different DMF : DMSO ratios (S1, S2, S3 and S4).

Fig. 6 presents the TRPL decay measurements of samples S1, S2, S3 and S4, showing a multi-exponential decay profile. The bi-exponential function (eqn (12)) is used to fit the TRPL decay comprising fast and slow components which correspond to defect-mediated non-radiative recombination at trap states and radiative band-to-band recombination, respectively.12
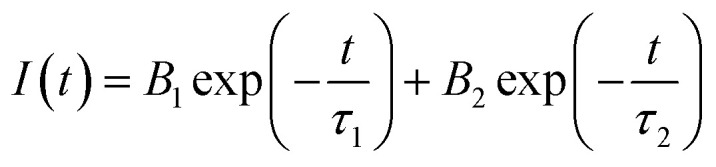


The average carrier lifetime derivates from TRPL decay measurements are presented in Table 2. The sample S3 exhibits the longest decay lifetime (*τ* = 845.9 ns) indicative of minimal non-radiative recombination. As confirmed by XRD and earlier optical analysis, this extended carrier lifetime results from efficient defect passivation at grain boundaries, where residual PbI_2_ compensates for iodide vacancies and under-coordinated Pb^2+^ defects. In contrast, S2 and S4 exhibit shorter PL lifetimes, indicative of recombination dominated by defects. A low amount of DMSO (sample S2) is insufficient to passivate defects leading to an increase in the trap density. On the other hand, an excessive amount (sample S4) induces the formation of amorphous PbI_2_ and unreacted DMSO residues which form deep-level traps. Therefore, an optimized intermediate DMF : DMSO ratio (6 : 4) provides the best balance between film uniformity, crystallinity, passivation, and charge transport properties, as supported by XRD, PL, and TRPL. These results underscore the delicate balance required in solvent engineering.

### 3.2 Modulization and simulation

**Fig. 7:**
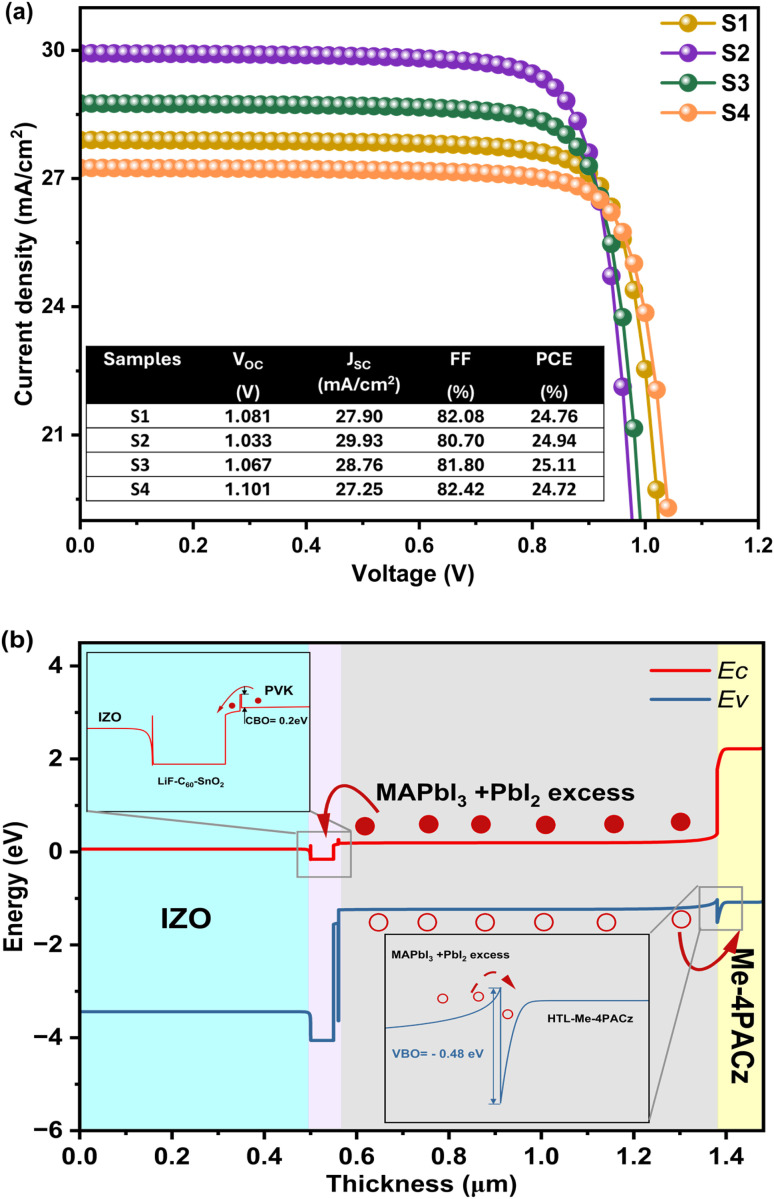
(a) *J*–*V* curves, for all samples. Inset showing a table summarizing the PV parameters. (b) The band diagram of one of the samples.

In this work, the experimentally obtained parameters (*τ*_avg_, thickness, and bandgap) of samples S1–S4 were implemented in SCAPS-1D simulations to model the performance of RGO/Me-4PACz/MAPbI_3_/LiF-C60-SnO_2_/IZO-structured PSCs. In the proposed structure, RGO was employed to enhance thermal stability, maintain PSC performance under high-temperature conditions, and improve device flexibility. On the other hand, IZO serves as an efficient back contact owing to its excellent optical transparency and electrical properties. Fig. 7 presents the impact of varying DMF : DMSO ratios (1 : 0, 8 : 2, 6 : 4, and 5 : 5) on the performance of perovskite solar cells under front and rear illumination, with all samples exhibiting the same excess of PbI_2_. For the sample S1 (1 : 0) and S3 (6 : 4), almost the same photovoltaic parameter are observed due to the poor film quality arising from the rapid evaporation because of presence of high ratio of DMF. Consequently, this last is evaporated rapidly leading to poor crystallization in the film. However, the sample S2 (8 : 2) improves the crystallization with the addition of DMSO, enhancing front illumination performance due to the better absorption and reduced defects. Interestingly, S4 (5 : 5) shows a reduced PL intensity reflecting a lower electron–hole recombination rate in the MAPbI_3_ films. Despite this improvement, slower crystallization may lead to residual defects that affect overall performance under rear illumination, although front illumination remains reasonable. These results indicate that front illumination is sensitive to perovskite film quality, while rear illumination highlights back contact passivation and light management. In conclusion, S3 achieves the most balanced performance and S4 shows promising reduction in recombination losses. In the next section, we will explore optimization strategies to enhance photovoltaic performance by selecting the appropriate HTL and ETL with optimal thickness, *E*_g_, defect density (*N*_t_), and parasitic resistances. The structural and optical results of MAPbI_3_ films prepared with different DMF : DMSO ratios, passivated the interface with PbI_2_ excess, proved that almost all these samples are suitable as absorbing layers in PCS, among them, S3 shows the best PV performance. In the next section, we will utilize S3 to find the optimal parameters by testing other ETLs, HTLs and TCO (back contact) to enhance PV performances.

**Fig. 8:**
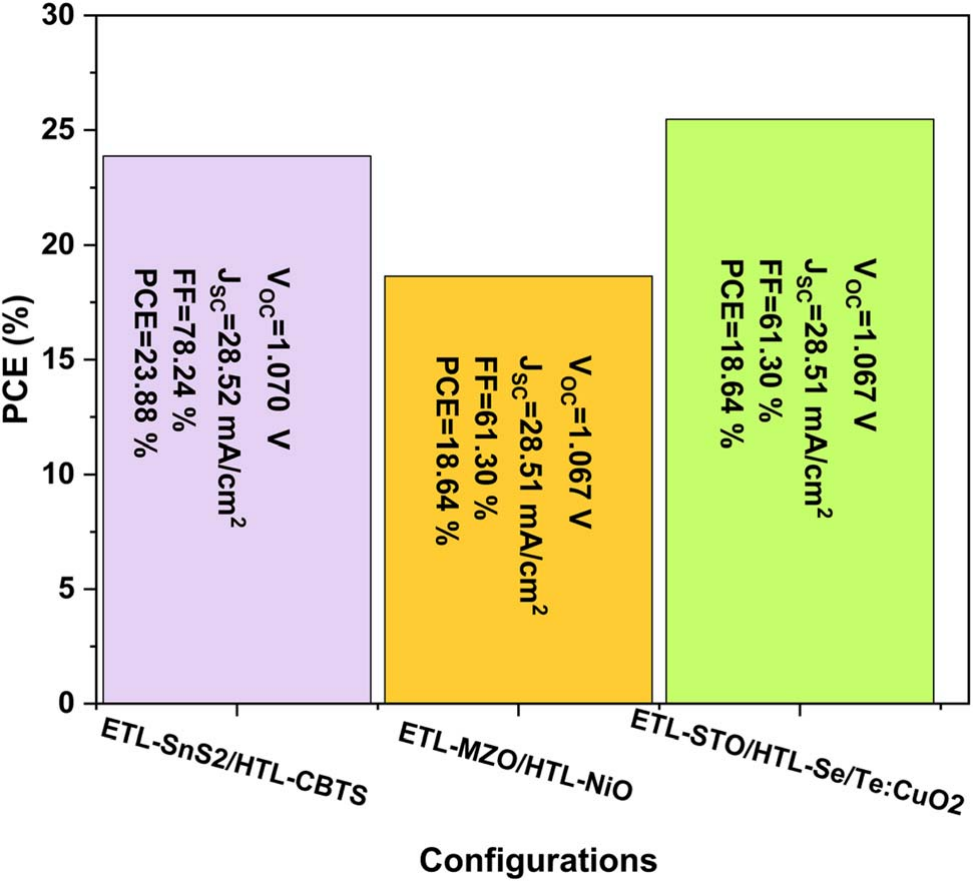
Histogram with PV performances of different configurations of PSCs.

In this subsection, the performance was analyzed using new HTL like CBTS, NiO, and Se/Te:CuO_2_, with ETL like SnS_2_, MZO, and STO (see Fig. 8). These carriers transporting layers result in the improvement of all PV performances where the PCE improved from 25.11% to 25.47%, *V*_OC_ from 1.067 V to 1.071 V, *J*_SC_ improved from 28.76 to 28.78 mA cm^−2^, and FF improved from 81.80% to 82.61%. These performances can be further optimized by investigating the effects of the thickness and defect density of the perovskite layer, which has the most significant impact on device performance.

**Fig. 9:**
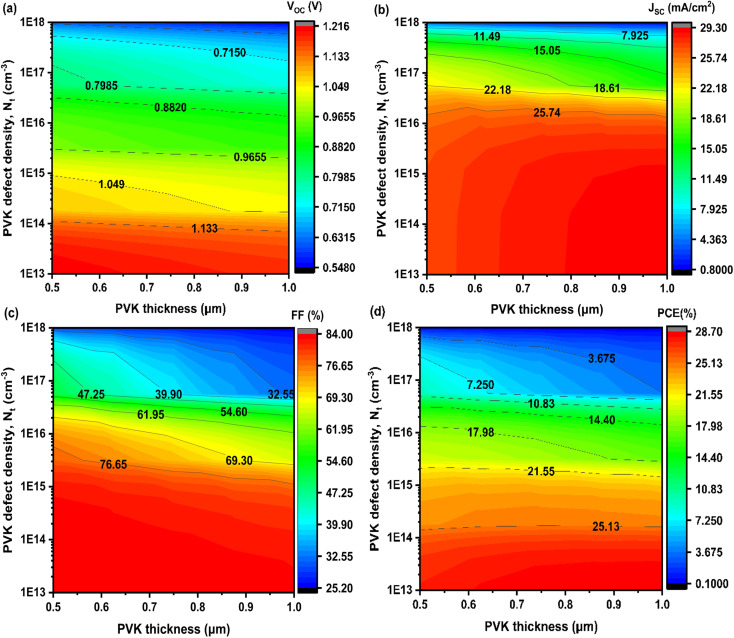
Contour plots of the effect of thickness and defect density (*N*_t_) of PVK layer on the PV performances.

The results depicted in Fig. 9 demonstrate a strong dependence of PV performance on both the thickness and defect density (*N*_t_) of PVK absorber layer. Specifically, increasing the PVK thickness from sub-micron levels up to 1 μm leads to notable improvements in PCE, *V*_OC_, *J*_SC_, and FF. This enhancement can be attributed to the improved light absorption and increased generation of photocarriers within the thicker absorber layer, which allows for more efficient photon harvesting across the solar spectrum.^50^ In contrast, increasing the defect density within the PVK layer has a detrimental effect on device performance. Higher values of *N*_t_ introduce more non-radiative recombination centers, which reduce carrier lifetime and increase recombination losses.^51^ These findings highlight the critical role of material quality in perovskite solar cells, emphasizing the need for optimized fabrication processes that minimize intrinsic and interfacial defect densities. The optimal performance is achieved at a PVK thickness of 1 μm and a defect density of 1 × 10^13^ cm^−3^, representing an ideal balance between efficient light absorption and reduced recombination losses. At these conditions, the device exhibits a PCE of 28.7%, *V*_OC_ of 1.216 V, *J*_SC_ of 29.30 mA cm^−2^, and FF of approximately 89%. Interestingly, variations in the thickness and defect density of the HTL, ETL, and TCO layers exhibit minimal influence on the overall photovoltaic performance.^52^ This indicates that once these layers fulfill their roles in charge transport and optical transparency, further optimization yields minimal gains. In contrast, the perovskite absorber layer has a dominant influence, as it directly governs light absorption and carrier generation.

**Fig. 10:**
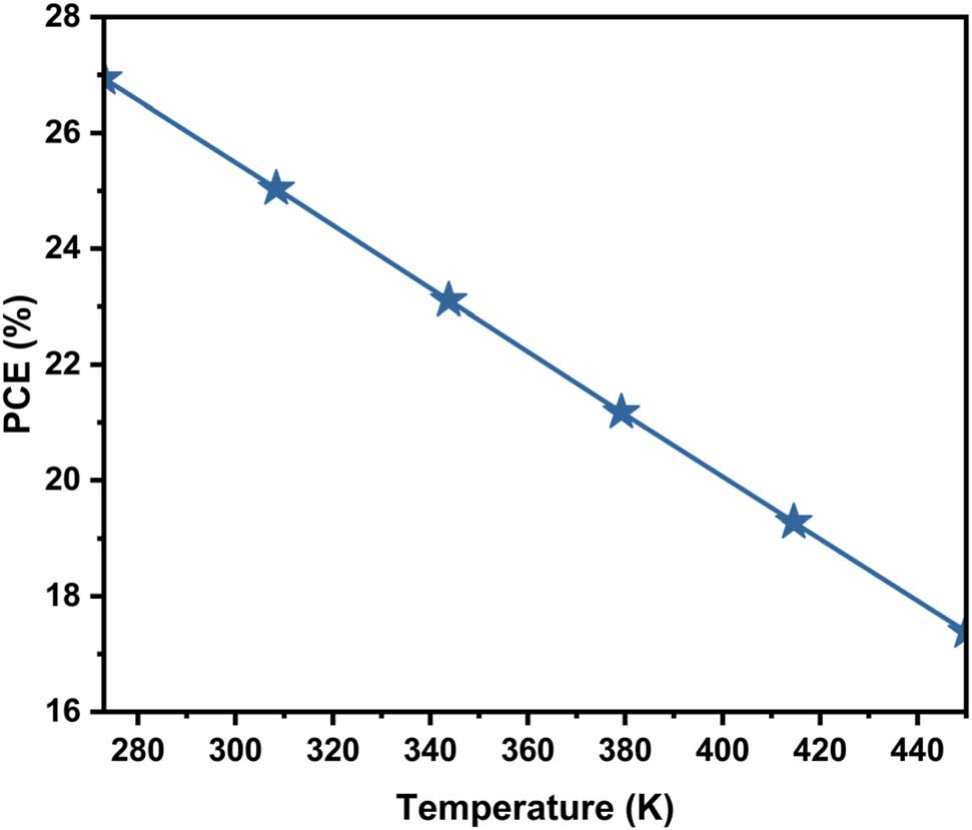
Influence of the temperature on the PCE of PSC.

The performance of the perovskite solar cell significantly declines as temperature increases from 275 K to 450 K, as illustrated in the Fig. 10. This reduction in PCE is primarily due to the thermally activated increase in non-radiative recombination, which lowers *V*_OC_ and FF. Therefore, ensuring thermal stability through material engineering and proper device encapsulation is essential for maintaining high performance in real-world operating conditions.

**Fig. 11:**
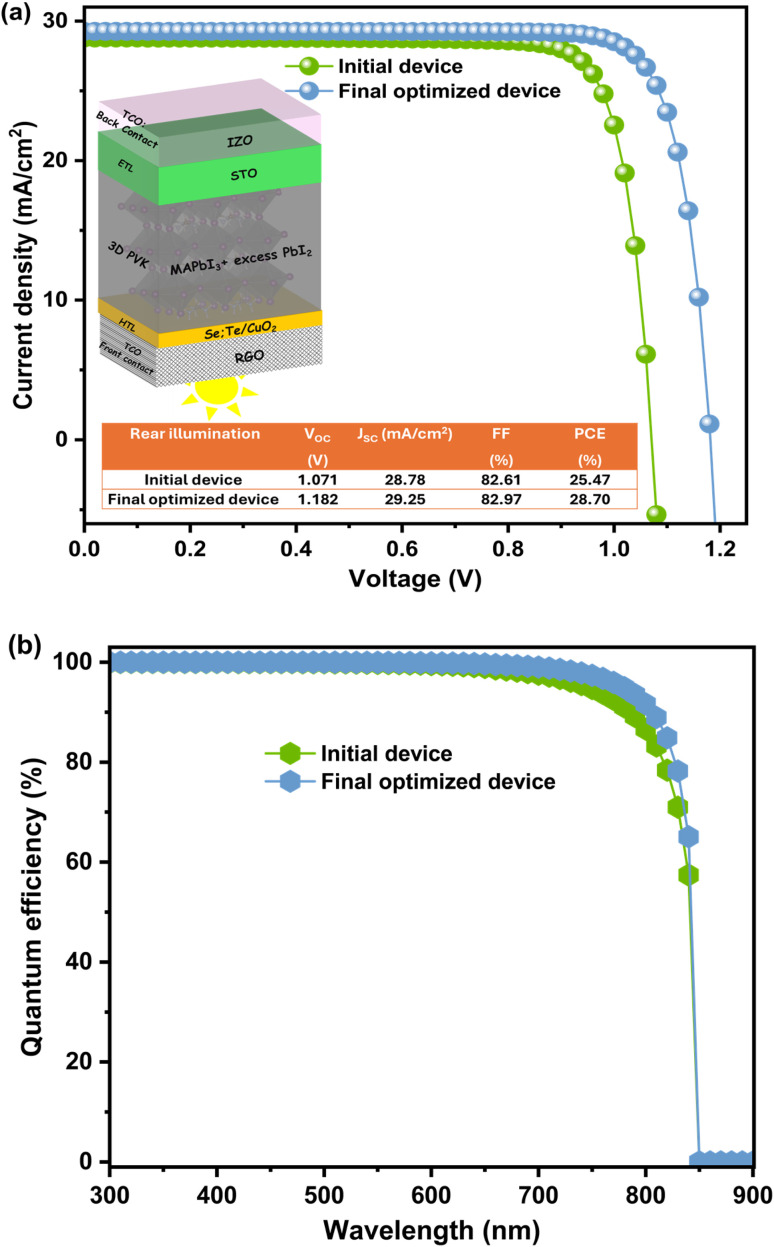
(a) *J*–*V* curves of initial and final optimized device with structure RGO/Se:Te/CuO_2_/MAPbI_3_ + PbI_2_ excess/STO/IZO. Inset presents structure and table summarizing all PV performance before and after optimization. (b) Quantum Efficiency (QE).

After applying the optimized MAPbI_3_ thickness and defect density, all photovoltaic parameters improved notably. The PCE increased from 25.47% to 28.70%, *V*_OC_ from 1.071 V to 1.182 V, *J*_SC_ from 28.78 to 29.25 mA cm^−2^, and FF from 82.61% to 82.97% (see Fig. 11(a)). These enhancements are mainly due to improved light absorption and reduced recombination losses. The optimal thickness ensures efficient photon harvesting, while the lower defect density suppresses non-radiative recombination, leading to better carrier collection and overall device performance. On the other hand, a significant improvement in quantum efficiency (QE), particularly within the 600 to 800 nm wavelength range, was observed after optimizing the thickness of MAPbI_3_ to 1 μm and lowest value the defect density, as presnted in Fig. 11b. This enhancement in QE can be attributed to the optimized material properties, which minimize carrier recombination, enhance light absorption, and improve charge transport efficiency.^53^ As a result, the device demonstrates better performance, especially in the critical visible range, contributing to its overall efficiency.

This section presents a comparison of our results with those from the literature, as summarized in Table 3, which includes key findings from previous works. Our device achieves superior performance, surpassing others in efficiency, suggesting that the use of MAPbI_3_ in inverted perovskite solar cells could lead to higher stability and enhanced efficiency. This opens up promising possibilities for the development of more stable and efficient solar cells in future applications.

**Table 3:** Comparison of the performance achieved with respect to previously reported works

Devices	*V*_OC_ (V)	*J*_SC_ (mA cm^−2^)	FF (%)	PCE (%)	Ref.
ITO/NiO_*x*_-p-F-PEAI/Cs_0.05_FA_0.85_MA_0.1_PbI_3_/C_60_/BCP/Cu	1.12	24.01	84.98	22.93	54
IZO/PTAA/perovskite/BHJ/Zr(acac)4/Ag	1.13	24.8	77.68	21.73	55
FTO/TiO_2_/IDL1/MAPbI_3_/IDL2/Se/Te:CuO_2_/Au	1.16	23.51	82.47	22.42	33
**RGO/Se:Te/CuO**_**2**_ **/MAPbI**_**3**_ **+ PbI**_**2**_ **excess/STO/IZO**	**1.18**	**29.25**	**82.97**	**28.70**	**This study**

## 4. Conclusion

This work investigates the interplay effect of the solvent engineering (DMF : DMSO ratios) and excess of PbI_2_ in optimizing the structural and optoelectronic properties of solution-processed perovskite films. Structural and optoelectronic analysis confirm that at ambient conditions a DMF : DMSO ratio of 6 : 4 promotes the formation of highly crystalline MAPbI_3_ films, with residual of PbI_2_, in addition to enhanced light harvesting efficiency accompanied by reduced Urbach energy (<20 meV), indicative of lower energetic disorder. Our findings emphasize the fundamental significance of solvent design and PbI_2_ management in perovskite film optimization, providing a model for future research bridging the gap between film quality and device performance, particularly for air-processed perovskite photovoltaics. SCAPS-1D simulations were conducted on the structure of RGO/Me-4PACz/MAPbI_3_/LiF-C_60_-SnO_2_/IZO, using experimental data to identify the optimal absorber. The simulations revealed that the sample with (6 : 4) as an DMF : DMSO ratio served as the best absorber, achieving an impressive power conversion efficiency (PCE) of 25.11%. Following this, a comprehensive optimization process was carried out by exploring various device configurations by incorporating novel hole transport layers such as NiO, CBTS, and Se/Te:CuO_2_, along with electron transport layers like SnS_2_, MZO, and STO. The analysis indicated that the optimized configuration of RGO/Se/Te:CuO_2_/MAPbI_3_/STO/IZO yielded the highest efficiency with a PCE increasing up to 28.70%. This significant improvement demonstrates the potential of solvent-engineered MAPbI_3_ for achieving high-performance perovskite solar cells. Our findings highlight the critical role of solvent tuning in balancing key factors such as crystallinity, defect passivation, and charge transport in ambient-air-processed perovskite films. By carefully optimizing these parameters in other similar perovskites, it is possible to enhance the stability and efficiency of perovskite-based solar cells, paving the way for the development of more efficient and durable solar technologies using other perovskite combinations.

## Conflicts of interest

There are no conflicts to declare.

## Data Availability

All data that support the findings of this study are included with the article.
